# Computational approaches to detect small lesions in ^18^F‐FDG PET/CT scans

**DOI:** 10.1002/acm2.13451

**Published:** 2021-10-13

**Authors:** Kenneth J. Nichols, Frank P. DiFilippo, Christopher J. Palestro

**Affiliations:** ^1^ Department of Radiology Donald and Barbara Zucker School of Medicine at Hofstra/Northwell Hempstead New York USA; ^2^ Department of Nuclear Medicine Cleveland Clinic Cleveland Ohio USA

**Keywords:** ^18^F, image analysis, oncology, PET, phantom simulations, radiomics

## Abstract

**Purpose:**

When physicians interpret ^18^F‐FDG PET/CT scans, they rely on their subjective visual impression of the presence of small lesions, the criteria for which may vary among readers. Our investigation used physical phantom scans to evaluate whether image texture analysis metrics reliably correspond to visual criteria used to identify lesions and accurately differentiate background regions from sub‐centimeter simulated lesions.

**Methods:**

Routinely collected quality assurance test data were processed retrospectively for 65 different ^18^F‐FDG PET scans performed of standardized phantoms on eight different PET/CT systems. Phantoms included 8‐, 12‐, 16‐, and 25‐mm diameter cylinders embedded in a cylindrical water bath, prepared with 2.5:1 activity‐to‐background ratio emulating typical whole‐body PET protocols. Voxel values in cylinder regions and background regions were sampled to compute several classes of image metrics. Two experienced physicists, blinded to quantified image metrics and to each other's readings, independently graded cylinder visibility on a 5‐level scale (0 = definitely not visible to 4 = definitely visible).

**Results:**

The three largest cylinders were visible in 100% of cases with a mean visibility score of 3.3 ± 1.2, while the smallest 8‐mm cylinder was visible in 58% of cases with a significantly lower mean visibility score of 1.5±1.1 (*P* < 0.0001). By ROC analysis, the polynomial‐fit signal‐to‐noise ratio was the most accurate at discriminating 8‐mm cylinders from the background, with accuracy greater than visual detection (93% ± 2% versus 76% ± 4%, *P* = 0.0001), and better sensitivity (94% versus 58%, *P* < 0.0001).

**Conclusion:**

Image texture analysis metrics are more sensitive than visual impressions for detecting sub‐centimeter simulated lesions. Therefore, image texture analysis metrics are potentially clinically useful for ^18^F‐FDG PET/CT studies.

**Abbreviations**
ANOVA: analysis of variance
FDG: ^18^F‐fluorodeoxyglucose
GLCM: gray‐level co‐occurrence matrix
PET: positron emission computed tomography
PSF: point spread function
QA: quality assurance
Q‐Q: quantile‐quantile plot
ROC AUC: receiver operating characteristics area under curve
ROI: region of interest
SNR: signal‐to‐noise ratio
SUV: standard uptake value

## 1 INTRODUCTION

When physicians interpret positron emission tomography/computed tomography (PET/CT) scans, they utilize standard uptake values (SUVs) of lesions,^1^ together with their visual impressions of the size and number of lesions. “Smaller lesions” are often meant to be those below 1 cm in diameter, and for some disease states lesions of any size require immediate intervention. The motivation for detecting small lesions is to discriminate evidence of disease from background image noise, as doing so can trigger a significant change in medical therapy.^2^ However, PET imaging has limited spatial resolution and is less sensitive and specific for smaller lesions.^3^, ^4^

Over the past several years, many approaches have been pursued to aid physicians in deciding whether a focus of radiotracer uptake is a genuine lesion or is due to random voxel value fluctuations of tissue background noise. Central to patient management is the discrimination of true small lesions from random image noise. This is complicated by the fact that although radioactive decay follows Poisson statistics, the voxel values in reconstructed PET/CT images do not. Several approaches to optimizing image quality are applied to PET/CT scans, including the use of nonlocal mean filtering,^5^ and adjusting reconstruction parameters to suppress background noise while selectively enhancing foci of activity.^6^

While some PET reconstruction neural network techniques reduce noise at the expense of image contrast, more recent deep learning refinements can decrease image noise and improve contrast simultaneously.^7^, ^8^ Deep learning methods are being applied to PET images for de‐noising, partial volume corrections,^9^ and scatter corrections.^10^, ^11^ Sophisticated neural networks, such as those that use dynamic PET data,^12^ and those that incorporate CT image information simultaneously with patient‐specific demographic and risk factor information, are helping detect small lung nodules.^13^ All of these approaches handle a great deal of input information simultaneously; some methods use only digital images as input,^14^ while other methods first extract radiomics features as input to neural networks to streamline input to neural networks,^15^ such as by selectively incorporating PET SUV information.^16^

Regardless of which approach is used to reconstruct PET images, there is a need to differentiate small lesions from background image noise. Image texture analysis can aid in identifying lung cancer and has been investigated as a means to distinguish between random noise in neighboring voxels versus a pattern of voxel values that are meaningfully correlated.^17^ This type of data analysis does not ask merely whether voxel values in an individual isolated voxel exceed the minimally detectable activity indicated by the voxel value levels of neighboring background voxels,^18^ but whether there is a discernable pattern of voxel values centered about a region. Clinicians do this in performing their visual analyses, but it has not been obvious which image texture analysis metrics may best reflect the confidence with which a clinician decides that a perceived PET scan voxel value fluctuation represents a genuine small lesion. Consequently, the challenge to be addressed is to determine whether there is an image texture analysis metric that is more accurate than visual judgments for differentiating a genuine lesion from noise in PET scans. Our investigation used physical phantom simulations to address two specific aims:

1. evaluate the agreement of quantitative texture analysis metrics with respect to visual interpretation, and
2. determine which image texture analysis metric best differentiates voxel values of background regions from those of sub‐centimeter lesions, regardless of which reconstruction methods have been applied to optimize PET/CT image quality.

## 2 MATERIALS AND METHODS

### 2.1 QA phantom

**FIGURE 1:**
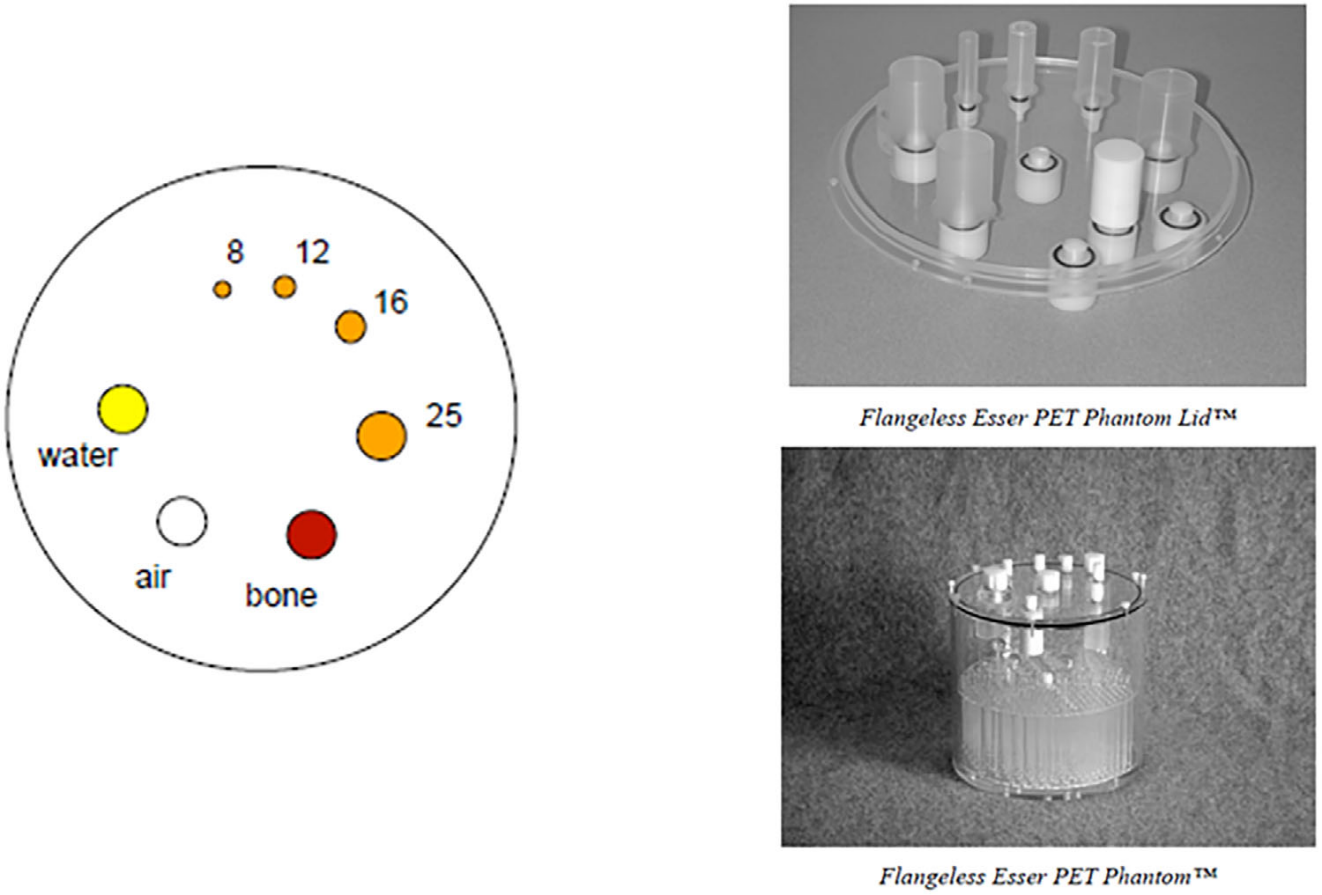
The standard PET phantom used for the PET/CT data acquistions

Data were examined retrospectively for 65 PET/CT phantom scans from eight different PET/CT systems acquired between 5 January 2016 and 3 January 2021. Activity concentrations were intended to produce a ratio of concentrations of 2.5:1 for “hot” cylinders to background,^19^ achieved with ∼13 kBq/mL for “hot” cylinder inserts and ∼5.2 kBq/mL for uniform background activity, prepared 60 min before the start of the PET acquisition, consistent with activity concentrations for typical whole‐body PET protocols for a 70 kg patient injected with 370 MBq (10 mCi) ^18^F‐FDG. These activity concentrations are recommended for PET system routine quarterly QA tests by the American College of Radiology (ACR),^20^ in conjunction with a standardized “flangeless Esser phantom,” which is a version of a “Jaszczak phantom.”^21^ This standardized phantom includes a ∼6 L cylindrical water bath, Plexiglas® inserts of six rod sizes in half the phantom, and seven cylinder inserts consisting of four “hot” cylinders of internal diameters 25 mm, 16 mm, 12 mm, and 8 mm, along with three “cold” inserts simulating bone, water, and air (Figure 1). As others have found it challenging to detect simulated 7‐mm spherical lesions on PET/CT scans unless target‐to‐background concentrations were > 4.0,^22^ discerning an 8‐mm cylindrical simulated lesion at 2.5:1 concentration ratio was deemed a reasonable goal in our investigation.

All phantom QA studies were acquired using routine clinical protocols for a whole‐body oncology PET/CT scan for a 70 kg adult male patient, as required by accrediting agencies, and reconstructed according to each manufacturer's recommendations. Six of the PET scanners were time‐of‐flight units (2 General Electric D710 systems, and Siemens Biograph 40‐mCT, 128‐mCT, 20‐mCT, and 64‐mCT) and 2 were not (Siemens Biograph 6 True Point and Biograph 40 True Point systems). Matrix sizes read from DICOM header files of reconstructed PET tomograms ranged from 168 × 168 pixels to 200 × 200 pixels with a mean pixel size of 3.8 ± 0.7 mm. By default, the mean reconstructed slice thickness was the same as the mean pixel size of 3.8 ± 0.7 mm. The CT scan was used for attenuation correction, which was implemented along with corrections for scatter and random events during reconstruction by iterative OSEM algorithms. Transaxial sections of each tomogram were normalized to have a maximum voxel value of 100 per pixel, and magnified to have a total diameter of 200 pixels out of 256 in order to regularize input data among the different PET systems. As all 3D voxels of each phantom data set were scaled by the same value per phantom, this normalization procedure preserved the relative noise characteristics of the data of each phantom.

### 2.2 PET phantom tomographic section isolation algorithms

Algorithms were written in IDL v 8.4 (Harris Geospatial Solutions, Broomfield, CO) to automatically process routinely acquired PET phantom data in accordance with standard laboratory accreditation procedures. The algorithms automatically determined optimal transaxial slice locations for “hot” cylinders, rods, and uniform PET phantom volumes. These algorithms were applied to DICOM (NEMA) data that were transferred to a standalone PC running Windows 10 (Microsoft Corp, Redmond WA). The location within transaxial sections and the sequence from largest to smallest cylinders and rods were automatically determined.

**FIGURE 2:**
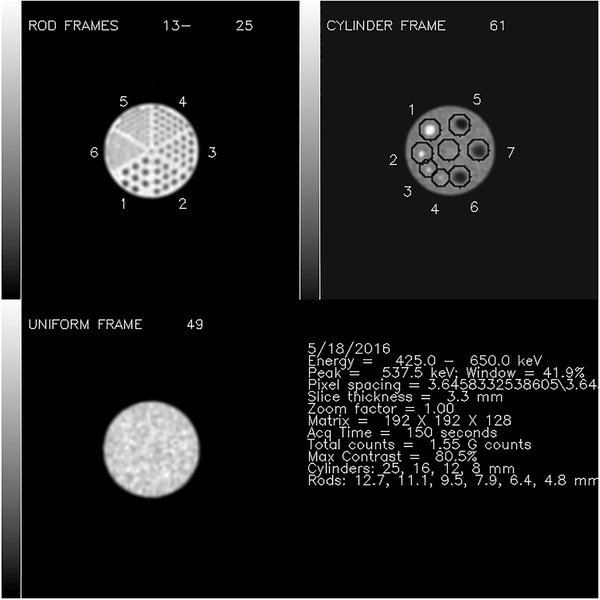
A summary screen reporting the automatically generated QA report for a standard quarterly PET/CT data acquisition

**FIGURE 3:**
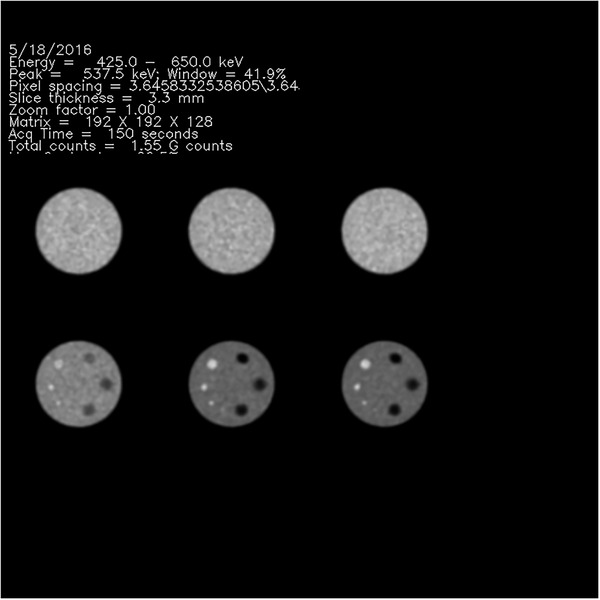
One of the jpg files of the automatically generated QA report for a standardized PET/CT data acquisition, which was used for the visual scoring of confidence of “hot” cylinders visibility

The maximum “hot” cylinder voxel value identified the single 3.8 ± 0.7 mm‐thick transaxial slice selected for the generation of the “hot” insert regions of interest (ROIs). Two‐dimensional (2D) ROIs in the single transaxial tomographic section that passed through the most intense pixel were generated automatically for each cylinder, and an identically sized background ROI in the center of the slice, for a total of eight ROIs (Figure 2). The automated ROI algorithm placed the centers of the “hot” cylinders away from the location of the maximum voxel value of the hottest cylinder by predetermined angles, but allowed for the possibility that the brightest voxel within a “hot” cylinder could be offset from the expected center, and relocated the center based on the location of each “hot” cylinder's actual tabulated maximum voxel value. Each ROI was generated to have a 40‐mm diameter, so that each of the eight ROIs was larger than the maximum cylinder diameter of 25 mm (Figure 1). A summary .jpg image was generated (Figure 2), along with jpg files showing all reconstructed PET phantom transaxial sections summed into 1‐cm‐thick slices (Figure 3). While all algorithms were automated, provisions were made to alter transaxial slice locations and ROI centers if necessary. Because it was possible that the center of the ROI could be incorrectly identified with a random maximum voxel value fluctuation, the center of each of the automatically generated ROIs was carefully examined visually by the same medical physicist for each instance. In cases for which it was not possible to verify visually an 8‐mm ”hot” insert, the ROI was drawn manually, centered in the vicinity of the a priori known location of the 8‐mm ”hot” insert, and of a diameter similar to the automatically generated ROIs.

### 2.3 Image metrics

Four classes of image characteristics metrics were investigated: (1) curve fitting metrics; (2) voxel value quantile curve metrics; (3) gray‐level co‐occurrence matrix (GLCM) metrics, and (4) voxel value histogram metrics.

Curve fitting was investigated based on the a priori knowledge that voxel values of a lesion smaller than twice the spatial resolution should follow an organized pattern of decreasing values with increasing distance away from the lesion's geometric center, that is, similar to the system point spread function (PSF).^23^ Curve fitting was applied to the voxel values tabulated for each of the “hot,” “cold,” and background ROIs. As the simulated lesions in the phantom were cylinders, not spheres, we performed curve fitting of voxel values sampled in a single 2D transaxial section instead of performing a 3D curve fit to the voxel values of a series of stacked neighboring 2D transaxial sections.

Quantile curve, GLCM, and histogram analyses potentially can detect any deviation from a random number distribution, and have been studied previously in relation to detecting cold spheres in SPECT phantoms.^24^

#### 2.3.1 Curve fitting

**FIGURE 4:**
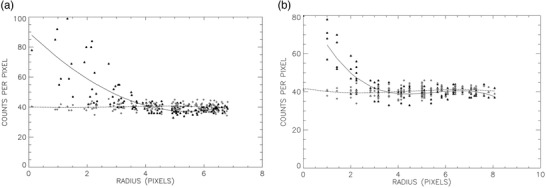
Plots of the 16‐mm (a) and 8‐mm (b) cylinder voxel values (black triangles) and background voxel values (gray diamonds) versus radii along with the polynomial‐fitted curves (solid curves for cylinders; dashed curves for background values)

For automatically generated ROIs, locations of voxel values within the ROI were transformed into polar coordinates centered on the maximum voxel value in the ROI. If the reader felt it was necessary to manually alter an ROI, such as for the 8‐mm cylinder, then locations of voxel values were transformed into polar coordinates centered on the center of the manually created ROI. To generate a third order polynomial fit on the voxel values (F) versus radii in units of pixels (r) measured outward away from the location of the central maximum pixel value (Figure 4), polynomial curve‐fitting algorithms included with the IDL programming language were employed:

(1) $$F\left(r\right)=F_{0}+F_{1}r+F_{2}r^{2}+F_{3}r^{3}$$

where F_0_ is the constant fitting parameter at the center of the ROI, and F_N_ are the coefficients for each of the N powers of radius r. The IDL least‐squares polynomial‐fitting algorithms used matrix inversion to generate both the fitting constants F_N_ and the standard deviation of each of the F_N_ fitting constants (σ(F_N_)),^25^ which were determined empirically by the data noise. Non‐linear least‐squares curve fitting was applied for up to 20 successive iterative estimates of fitted curve values obtained from altering fitting parameters and compared with each iteration to the χ^2^ measure of fitted points versus input data points, until convergence was achieved with a χ^2^ difference from one iteration to the next of tolerance of <10^−3^. Changes in fitting parameters between iterations were guided by a gradient‐expansion algorithm.^25^ The polynomial‐fitted contrast was computed as:

(2) $$\begin{array}{ccc} & & \mathrm{Polynomial}-\text{fit}\;\text{contrast}\;\\ & & \;=\left(F_{0}-F\left(r_{\max}\right)\right)/\left(F_{0}+F\left(r_{\max}\right)\right) \end{array}$$

where F_0_ is the intercept and F(r_max_) is the value of the fitted curve at the maximum radius. The rationale for fitting terms to polar coordinates is that there should be a pattern of ascending counts with decreased radii toward the center of a lesion, which should reinforce at all angles; a search for a converged fit to the radial counts around the lesion center will yield a fitting value F_0_ at the center that is distinguishably greater than the mean background count far from the center F(r_max_)). If there is no lesion, then F_0_ is expected to be equal to the mean background, and F_N_ is expected to be zero for N = 1, 2, and 3.

To compare the polynomial‐fit contrast of the simulated lesions to contrast in uniform background regions, we performed a polynomial fit for voxel values sampled in the ROI of the central uniform phantom volume, centered on the local maximum, the same as for the “hot” insert ROIs, assuming the center of the ROI to correspond to the center of a possible lesion. For the voxel values tabulated within these uniform volume ROIs, we also computed the conventional “raw” image contrast (IC) from maximum and minimum voxel values for each sampled background voxel value ROI as:

(3) $$\text{Raw}\;IC_{\mathrm{Background}}=\frac{\mathrm{maximum}-\mathrm{minimum}}{\mathrm{maximum}+\mathrm{minimum}}$$

The polynomial fit signal‐to‐noise (SNR) ratio also was computed, as:

(4) $$\mathrm{Polynomial}-\mathrm{fit}\mathrm{SNR}={\left(F_{0}/\sigma \left(F_{0}\right)\right)}^{2}$$

Note that σ(F_0_) was the computed uncertainty in the value of the curve‐fitting component F_0_, not the uncertainty in uniform background voxel value estimates, so that “Polynomial‐fit SNR” is not identical to conventionally defined signal‐to‐noise, which instead uses the uncertainty in background counts.^26^

**FIGURE 5:**
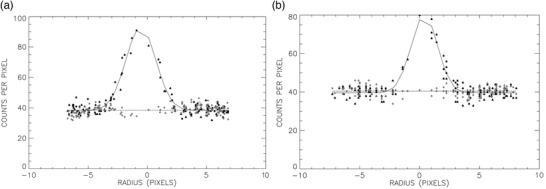
Plots of the 16‐mm (a) and 8‐mm (b) cylinder voxel values (black triangles) and background voxel values (gray diamonds) versus radii along with the Gaussian‐fitted curves (solid curves for cylinders; dashed curves for background values)

The data tabulated for each 2D ROI were also fit to Gaussian functions using IDL‐supplied subroutines (Figure 5), except that radii (r) were classified as positive for the right of center and negative if left of center of the location of the maximum voxel value in the ROI:

(5) $$G\left(r\right)=G_{0}*\exp\left(-{\left(\left(r-G_{1}\right)/G_{2}\right)}^{2}\right)+G_{3}$$

where G_0_ is the constant fitting parameter at the center of the ROI, G_3_ is the background value, G_2_ is the full width at half maximum in units of pixels, and G_1_ is the offset from 0‐radius. With these fitting constants, Gaussian‐fit contrast was computed as:

(6) $$\mathrm{Gaussian}-\mathrm{fit}\mathrm{contrast}=\left(G_{0}-G_{3}\right)/\left(G_{0}+G_{3}\right)$$

and the Gaussian‐fit SNR was computed as:

(7) $$\mathrm{Gaussian}-\mathrm{fit}\mathrm{SNR}={\left(G_{0}/\sigma \left(G_{0}\right)\right)}^{2}$$

as the IDL Gaussian‐fitting algorithms also computed standard deviations of the fitting parameters. As with the polynomial‐fit SNR (equation 4), we used the σ(G_0_) for the computed uncertainty in the value of the component, not the uncertainty in uniform background voxel value estimates, so that “Gaussian‐fit SNR” is different from the usual definition of signal‐to‐noise. The same Gaussian fitting algorithms also were applied to the tabulated voxel values of the uniform ROIs for comparison.

We also computed a Gaussian‐fitted integral from these fitting parameters as:

(8) $$\text{Gaussian}\;\text{integral}\;=\left(G_{0}-G3\right)*G_{2}$$

Based on the concept that if data can be successfully fit to a Gaussian function, then both curve “height” G_0_ above mean background G_3_ and curve “width” G_2_ should be meaningful positive definite numerical values. This integral should be a reasonable approximation of the sum of all lesion voxel values above and beyond background voxel values of a similarly sized phantom volume.

The IDL polynomial fitting and Gaussian fitting algorithms reported standard error of the estimate (SEE) and χ^2^ goodness of fit values, and indicated whether it was possible to converge successfully on a solution. If the fit was not successful, then the fitting parameters did not converge to a solution and values were undefined, in which case all metrics were set to 0. Ratios of fitting errors to fitting coefficients were computed to gauge “goodness of fit” for both polynomial‐fitting and Gaussian‐fitting solutions.

#### 2.3.2 Quantile curves

**FIGURE 6:**
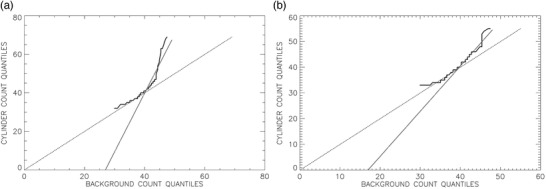
Quantiles of cylinder voxel values plotted as the darkest curve versus quantiles of background voxel values for the 16‐mm (a) and 8‐mm (b) cylinders. The dotted line is the line of identity. The dotted‐dashed line is the least‐squares fit to the upper 50% of cylinder voxel values

Voxel value quantile plots are one means of discerning significant deviations from random voxel value distributions.^27^, ^28^ Quantile‐quantile (Q‐Q) plots were created by graphing quantiles of tabulated voxel values of each “hot” insert ROI, sorted from minimum to maximum, against quantiles of minimum to maximum voxel values of background ROIs (Figure 6). A Q‐Q plot of voxel values sampled in one uniform background ROI should lie along the line of unity when plotted against voxel values of any other uniform background ROI. Linear regression was applied to the upper half of the Q‐Q curves, because that is the realm in which “hot” insert voxel values should exceed background values if they are greater than median background values. Statistically significant deviations from the line of unity for Q‐Q curves of least‐squares‐fitted slopes or intercepts were considered as evidence of detected “hot” inserts.

#### 2.3.3 GLCM metrics

GLCM matrices M(*i*, *j*) were formed, which tabulated the number of times a grayscale voxel value level *i* co‐occurred with voxel value level *j* within a 1‐pixel 2‐dimensional neighborhood.^29^ Construction of these matrices enabled the computation of a variety of conventional image texture analysis metrics, including GLCM Energy (a measure of orderliness), GLCM Entropy (a measure of randomness),^30^, ^31^ GLCM Inertia,^32^ (sometimes referred to in the literature as GLCM contrast),^33^ GLCM Homogeneity (a measure of regional dissimilarity), and GLCM Correlation. Each of the GLCM metrics was normalized to the highest value of that metric among the “hot” insert and background ROIs calculated for a given phantom.

#### 2.3.4 Histogram metrics

**FIGURE 7:**
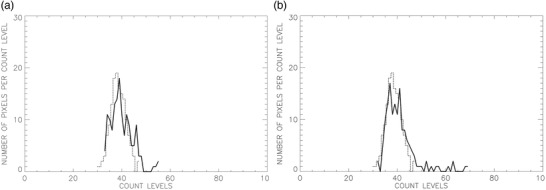
Voxel value histogram plots for 16‐mm (a) and 8‐mm (b) cylinders and background voxel values. Solid curves represent cylinder voxel values and dashed curves represent background

Phantom voxel value histograms were assessed to determine if values were normally distributed (Figure 7). Histogram metrics included curve means, variance, skewness, and kurtosis, which have been investigated in relation to oncologic PET studies.^34^ The rationale for tabulating these parameters was to determine if any of these measurements were useful for differentiating voxel values derived within a “hot” insert ROI from voxel values derived within a comparably sized background ROI.

Computed voxel value histogram metrics included maximum and mean SUV values. These maximum and mean SUV values were computed in a straightforward manner as the ratios of the maximum and mean voxel value per pixel in a 2D ROI to the central background voxel value per pixel.

### 2.4 Visual analysis

To gauge inter‐observer agreement, two medical nuclear physicists, both with more than 20 years’ experience, viewed the jpg summary files generated by the automated algorithms (Figure 3), independently of one another and without knowledge of computed image metrics. They scored their confidence of “hot” insert visibility on a 5‐level scale: 0 = “definitely not visible,” 1 = “probably not visible,” 2 = “equivocal,” 3 = “probably visible” and 4 = “definitely visible.” They also assigned dichotomous visibility judgments to each “hot” insert. One of the physicists rescored all phantom images a second time, blinded to his previous scores and other data, to assess intraobserver reproducibility. Both readers also were asked to assign a score to the background region as > 0 if they perceived that the magnitude of a random voxel value fluctuation in the background ROI was at least as intense as that within the neighboring 8‐mm insert ROI.

### 2.5 Statistical analysis

Statistical analyses were performed using commercially available “MedCalc” software.^35^ Values are reported as means ± one standard deviation. Visual reading interobserver agreement and intraobserver reproducibility were assessed by the kappa statistic of inter‐rater agreement, for which strength of agreement is considered “poor” for *κ* < 0.20, “fair” for *κ* = 0.21‐0.40, “moderate” for *κ* = 0.41‐0.60, “good” for *κ* = 0.61‐0.80, and “very good” for *κ* ≥ 0.81.^36^ Continuous variables were assessed by the χ^2^ test to determine the normality of distributions. ANOVA assessed whether there were differences between categories of continuous variables. The unpaired or paired *t*‐test, as appropriate, compared values between groups for continuous variables that were normally distributed; otherwise, the Mann‐Whitney or Wilcoxon test was used. χ^2^ analysis of proportions compared ratios. ROC analysis established optimal discrimination thresholds using dichotomous visual readings, and for discriminating 8‐mm insert regions from background voxel value regions. ROC analyses measured sensitivity, specificity, and accuracy (ROC area under curve, AUC) for each metric. For all tests, probability (*P*) < 0.05 was defined as statistically significant.

## 3 RESULTS

### 3.1 Data characterization

Following image reconstruction, there typically were 10^9^ total phantom voxel values, which were not normally distributed (χ^2^ *P* < 0.0001). Voxel values sampled in uniform volumes of phantoms had significant positive kurtosis (0.1 ± 1.4) ranging up to 11.0, indicating a narrow range of voxel values distributed about the mean value of 42 ± 5 counts per pixel for 109–145 pixels sampled per ROI, depending on data acquisition pixel size. For the 25, 16, 12, and 8‐mm cylinders maximum SUV values were 2.4 ± 0.3, 2.3 ± 0.3, 1.9 ± 0.3, and 1.4 ± 0.2, respectively, while mean SUV values were 1.2 ± 0.1, 1.1 ± 0.1, 1.0 ± 0.1, and 1.0 ± 0.1. Background maximum SUV and mean SUV values were 1.3 ± 0.1 and 1.0 ± 0.02.

### 3.2 Algorithm success rate

The localization algorithms correctly identified the slice visually confirmed to be optimal for visualization of the “hottest” cylinders in 64 of 65 (98%) cases; the algorithms succeeded in identifying the appropriate rods and uniform sections in 100% (65/65) of cases. (Figure 2). The locations of the ROIs for the 12–25 mm cylinders were successfully localized 100% of the time, but it was often necessary to adjust the location of the center of the 8‐mm cylinder ROI, because the automatically suggested 8‐mm ROI was too far from the known cylinder location (Figure 1).

The IDL polynomial curve fitting routines converged successfully for all simulated lesions and all background count samples for all phantoms. For polynomial‐fits, ANOVA demonstrated no difference between uniform, 8‐mm, and 12‐mm simulated lesion SEE (4‐5%), and no difference between 16‐mm and 25‐mm simulated lesion count SEE (7‐8%). There was a significantly lower (*P* < 0.001) SEE for uniform, 8‐mm, and 12‐mm simulated lesions compared to 16‐mm and 25‐mm simulated lesions. The results were similar for uniform, 8‐mm, and 12‐mm simulated lesion compared to 16‐mm and 25‐mm simulated lesions χ^2^ goodness of fit results (20‐40 versus 70–80). That is expected, as the magnitude of the range of counts in the larger simulated lesions is greater than the range of counts sampled for the smaller simulated lesions.

For Gaussian‐fits, we found a similar pattern of results for both SEE and χ^2^ goodness of fit measures, for the cases that converged. However, the Gaussian fitting routines were not as robust as the polynomial‐fitting routines; they failed to converge within 20 iterations with χ^2^ difference tolerance of 10^−3^ for the 8‐mm simulated lesion in 5 of the 65 phantoms, although they did converge for all larger simulated lesions. For the 8‐mm simulated lesions that did have curve fitting convergence, ratios of fitting errors to fitting coefficients were lower for cases that were deemed visible than those that were not for polynomial fits (18 ± 11% versus 40 ± 38%, *P* = 0.001) and for Gaussian fits (16 ± 6% versus 20 ± 7%, *P* = 0.03). The Gaussian χ^2^ goodness of fit values for the 8‐mm lesions were higher for the five cases in which Gaussian fitting failed to converge compared to the 60 cases in which it did converge (24 ± 11 versus 13 ± 7, *P* = 0.002), while the polynomial‐fitting χ^2^ goodness of fit values were the same for both groups (*P* = 0.53). For the five cases in which Gaussian fitting did not converge, the visual scores were ≤1, indicating that the readers judged these to be invisible. The Gaussian‐fitting algorithms failed to converge when applied to the uniform background count distributions in 13% of the cases. Consequently, the failure of the Gaussian fitting algorithms to converge was consistent with the absence of a significant signal in sampled count data.

### 3.3 Visual scores

Kappa values were nearly identical for inter‐reader and intra‐reader comparisons. Agreement between readers was “good” (*κ* = 0.77) for all 5‐level cylinder readings and “good” for dichotomous readings (*κ* = 0.80), but significantly lower (*P* < 0.0001), and only “moderate” for the subgroup of 5‐level 8‐mm cylinder readings (*κ* = 0.45), and “moderate” for dichotomous readings (*κ* = 0.41).

Similarly, intra‐reader reproducibility was “good” (*κ* = 0.76) for all 5‐level cylinder readings and “good” for dichotomous readings (*κ* = 0.78), but significantly lower (*P* < 0.0001), and only “moderate” for the subgroup of 8‐mm cylinder readings (*κ* = 0.49), and “moderate” for dichotomous readings (κ = 0.43).

### 3.4 Cylinder visibility

The three largest cylinders were visible in 100% of cases with a mean visibility score of 3.3 ± 1.2. The mean score was >1, and therefore considered visible, for 58% (38/65) of the 8‐mm cylinders, with significantly lower scores (*P* < 0.0001) than for the 12–25 mm cylinders but significantly higher than background (1.5 ± 1.1 versus 0.5 ± 0.5, *P* < 0.001). Mean background scores were visible (>1) in five of 65 of the phantoms, a false positive rate of 8%.

**FIGURE 8:**
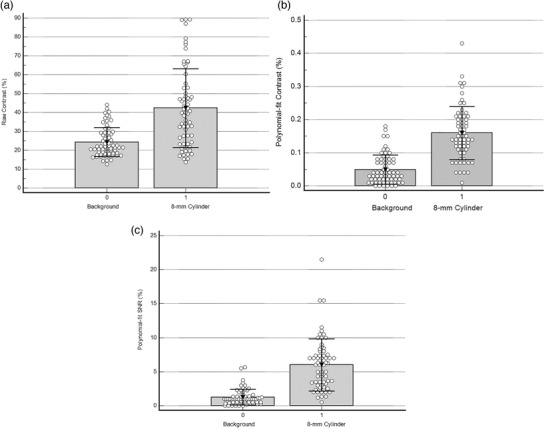
Comparison of metrics between background regions and 8‐mm cylinders for (a) raw contrast, (b) polynomial‐fit contrast, and (c) polynomial‐fit SNR

The Q‐Q intercept and Q‐Q slope, polynomial‐fit contrast and histogram skewness all performed equally well (ROC AUC = 97±1%) in agreeing with the cylinders and background regions that were marked as visible (Table 1). For correctly identifying the actual cylinders from background regions for all cylinder sizes, polynomial‐fit contrast and polynomial‐fit SNR were most accurate, and significantly more sensitive than the Q‐Q intercept or Q‐Q slope (Table 2). All of the other computed image texture metrics had ROC AUC values that were below those shown in Tables 1, 2. Background raw contrast was 24±8% instead of 0% (Figure 8a), while polynomial‐fit background contrast was 5 ± 4% (Figure 8b; Table 2). This is because the computation of raw background contrast is based on selectively finding the maximum and minimum counts from within a sample of background counts, and these values reflect the distribution of counts. While our counts were not normally distributed, a greater percentage of noise‐to‐signal is expected as the mean count decreases, yet computation of raw contrast makes no allowance for noise in the data. For a mean background count of 42±5 counts for 109–145 sampled pixels, one expects some pixels would have values up to 2 standard deviations above and below the mean, consistent with the observed mean raw background contrast of 24%. Similarly, background maximum SUVs were 1.3 ± 0.1 instead of 1.0 (Table 2). Minimally detectable signals are considered to be those for which a measurement exceeds 3 standard deviations,^18^ so that the mean SUV value of 1.3 above an SD of 0.1 indicates a significant chance of error in falsely identifying random background noise as a genuine lesion in the phantom images that we analyzed. This further illustrates the limitations of basing these metrics on maximum voxel values that are subject to noise fluctuations.^37^

**TABLE 1:** ROC for agreement with cylinder visibility for all cylinder sizes and ANOVA of visible versus not visible cases

Parameter	AUC (N = 325)	Sensitivity (N = 238)	Specificity (N = 87)	ROC Threshold	Visible (N = 238)	Not visible(N = 87)
Q‐Q intercept	97 ± 1%	90%	97%	<‐27	‐155 ± 99**	0 ± 14
Q‐Q slope	97 ± 1%	87%	99%	>1.8	4.8 ± 2.6**	1.0 ± 0.3
Polynomial‐fit contrast	97 ± 1%	88%	97%	>18%	34 ± 12%**	7 ± 6%
Histogram skewness	97 ± 1%	90%	98%	>0.9	1.8 ± 0.8%**	0.2 ± 0.3
Maximum SUVs	96 ± 1%	90%	94%	>1.41	2.0 ± 0.4**	1.3 ± 0.1
Polynomial‐fit SNR	95 ± 1%*	91%	90%	>4.3	15.3 ± 9.7**	2.1 ± 2.0
Gaussian‐fit integral	94 ± 1%*	84%	94%	>18.6	65.6 ± 47.0**	4.9 ± 7.6
Gaussian‐fit SNR	94 ± 1%*	81%*	98%	>7.9	17.5 ± 11.2**	2.4 ± 2.8
Raw contrast	90 ± 2%*	92%	79%*	>33%	47 ± 11%**	27 ± 15%

Abbreviations: AUC, area under curve; ROC, receiver operating characteristics; ANOVA, analysis of variance; Q‐Q, voxel value quantiles plots; SUV, standard uptake value; SNR, signal‐to‐noise ratio.

* *P* < 0.05 versus Q‐Q intercept.

** ANOVA *P* < 0.001 versus Not visible.

**TABLE 2:** ROC results for discriminating cylinders of all sizes from background and ANOVA of visible versus not visible cases

Parameter	AUC (N = 325)	Sensitivity (N = 260)	Specificity (N = 65)	ROC Threshold	Cylinder (N = 260)	Background (N = 65)
Polynomial‐fit contrast	97 ± 1%	92%	92%	>11%	33 ± 13%**	5 ± 4%
Polynomial‐fit SNR	97 ± 1%	90%	95%	>3.3	14.4 ± 9.8**	1.2 ± 1.6
Gaussian‐fit integral	95 ± 1%*	88%	95%	>10.9	61.2 ± 47.4**	2.1 ± 5.3
Gaussian‐fit SNR	94 ± 1%*	88%	92%	>4.7	16.4 ± 11.3**	1.7 ± 2.3
Q‐Q intercept	94 ± 1%*	83%*	100%*	<‐27	–142 ± 107**	1 ± 13
Q‐Q slope	94 ± 1%*	83%*	100%*	>1.7	4.4 ± 2.7**	1.0 ± 0.3
Histogram skewness	94 ± 1%*	83%*	100%*	>0.9	1.7 ± 0.9%**	0.2 ± 0.3
Raw contrast	93 ± 2%*	90%	83%	>32%	46 ± 13%**	24 ± 8%
Maximum SUVs	92 ± 2%*	79%*	99%	>1.48	2.0 ± 0.5**	1.3 ± 0.1

Abbreviations: AUC, area under curve; ROC, receiver operating characteristics; ANOVA, analysis of variance; Q‐Q, voxel value quantiles plots; SUV, standard uptake value; SNR, signal‐to‐noise ratio.

* *P* < 0.05 versus Polynomial‐fit contrast.

** ANOVA *P* < 0.001 versus Background.

Fifty‐eight percent (38/65) of the 8‐mm cylinders and 8% (5/65) of the background regions had mean scores > 1 while 42% (27/65) of 8‐mm cylinders and 92% (60/65) of background regions had mean scores ≤ 1. In terms of agreement with reader's scores, Q‐Q intercept and Q‐Q slope were the most accurate (ROC AUC = 87±3%), but were not significantly more accurate than the other metrics that are listed in Table 3, including polynomial‐fit contrast and polynomial‐fit SNR.

**TABLE 3:** ROC results for agreement with visibility of 8‐mm cylinders

Parameter	AUC (N = 130)	Sensitivity (N = 43)	Specificity (N = 87)	ROC Threshold	Visible (N = 43)	Not visible (N = 87)
Q‐Q intercept	87 ± 3%	74%	87%	<–16	–24 ± 16**	0 ± 14
Q‐Q slope	87 ± 3%	81%	81%	>1.2	1.6 ± 0.4**	1.0 ± 0.3
Polynomial‐fit SNR	86 ± 4%	72%	90%	>4.3	6.8 ± 4.3**	2.0 ± 2.0
Polynomial‐fit contrast	85 ± 4%	79%	79%	>11%	18 ± 9%**	7 ± 6%
Raw contrast	83 ± 4%	84%	70%*	>28%	45 ± 18%**	27 ± 15%
Maximum SUVs	82 ± 4%	86%	66%*	>1.3	1.4 ± 0.2**	1.3 ± 0.1
Histogram skewness	82 ± 4%	72%	89%	>1.3	0.8 ± 0.6**	0.2 ± 0.3
Gaussian‐fit SNR	81 ± 4%	84%	74%	>3.6	5.8 ± 3.5**	2.4 ± 2.8
Gaussian‐fit integral	80 ± 4%	79%	77%	>9.6	14.8 ± 8.2**	4.9 ± 7.6

Abbreviations: AUC, area under curve; ROC, receiver operating characteristics; ANOVA, analysis of variance; Q‐Q, voxel value quantiles plots; SUV, standard uptake value; SNR, signal‐to‐noise ratio.

* *P* < 0.05 versus Q‐Q intercept.

** *P* < 0.001 versus not visible.

Our finding that was most directly relevant to the discrimination of sub‐centimeter lesions from random background noise in PET scans was that polynomial‐fit contrast and polynomial‐fit SNR were most accurate at correctly discriminating between 8‐mm “hot” cylinders and background, with ROC AUC = 90±3% and 93±2%, respectively (with dichotomous accuracy 85% and 83%, respectively), significantly more accurate than any of the other image metrics (Table 4). Polynomial‐fit SNR was also more sensitive (94%) than visual detection (58%, *P* < 0.0001) and maximum SUVs (69%, *P* = 0.0003; Table 4). It is notable that sensitivity to detect a simulated lesion that was actually present was higher for the polynomial‐fit contrast and polynomial‐fit SNR than for either visual analysis or SUVs (Table 4). Contrast computed from polynomial‐fit curves was lower for background and 8‐mm cylinders, and separated these by a wider margin (Figure 8b), than for raw contrast (Figure 8a). The separation between background and 8‐mm cylinder metric values was even more pronounced for the polynomial‐fit SNR metric (Figure 8c), which had the highest accuracy (93%) for discriminating between simulated 8‐mm lesions and background regions (Table 4).

**TABLE 4:** Discrimination of 8‐mm cylinders from background

Parameter	AUC (N = 130)	Sensitivity (N = 65)	Specificity (N = 65)	ROC Threshold	Cylinder (N = 65)	Background (N = 65)
Polynomial‐fit SNR	93 ± 2%	94%	77%	>1.6	6.0 ± 3.8**	1.3 ± 1.2
Polynomial‐fit contrast	90 ± 3%	77%*	89%	>10%	16 ± 8%**	5 ± 4%
Gaussian‐fit integral	84 ± 7%*	86%	83%	>5%	14.2 ± 8.0**	2.1 ± 5.3
Gaussian‐fit SNR	83 ± 4%*	79%*	85%	>3.6	5.4 ± 3.8**	1.7 ± 2.3
Raw contrast	78 ± 4%*	66%*	83%	>32%	42 ± 21%**	24 ± 8%
Q‐Q intercept	77 ± 4%*	58%*	88%	<‐15.6	–16 ± 19%**	1 ± 13%
Q‐Q slope	76 ± 4%*	65%*	82%	>112	1.0 ± 0.3**	1.4 ± 0.5
Visual	76 ± 4%*	58%*	92%	>1	1.5 ± 1.1**	0.5 ± 0.5
Histogram skewness	75 ± 4%*	62%*	86%	>0.4	0.6 ± 0.6**	0.2 ± 0.3
Maximum SUVs	67 ± 5%*	69%*	66%	>1.3	1.4 ± 0.2**	1.3 ± 0.1

Abbreviations: AUC, area under curve; ROC, receiver operating characteristics; ANOVA, analysis of variance; Q‐Q, voxel value quantiles plots; SUV, standard uptake value; SNR, signal‐to‐noise ratio.

* *P* < 0.05 versus polynomial‐fit SNR.

** ANOVA *P* < 0.001 versus Background.

## 4 DISCUSSION

Visual analysis and SUVs are the criteria typically used by clinicians to assess the disease. It is important for patient management to detect small lesions.^2^, ^4^ The results of our investigation suggest that this is a setting in which quantified image texture analysis metrics computed from voxel values extending over neighborhoods of voxels are more reliable than visual impressions and SUV values for detecting genuine small abnormalities. Since the smallest 8‐mm simulated lesions always were loaded with radioactivity, while the background phantom volumes always were uniform, the ability of any given data processing approach, such as polynomial curve‐fitting, to successfully identify a simulated lesion while expert readers cannot is a validation of the feasibility of using that particular data processing approach.

Increasing the acquisition time and administered activity improves the detection of small lesions with PET/CT, but there are practical limitations to this approach. Decreasing the size of reconstruction pixels,^20^ and improvements to reconstruction algorithms^17^ have been used to improve the detectability of small lesions. Measurement of SNR for phantom experiments that include small lesions can guide the adjustment of model parameters to optimize detection of small lesions.^6^, ^38^

In choosing among different reconstruction parameters in PET/CT scans, the clinician's visual impressions of the existence of lesions and SUVs often are the criteria on which such decisions are based.^39^, ^40^ For conventional PET/CT ^18^F‐FDG lung nodule scans, an SUV > 2.5 has been considered a trigger point to modify patient management,^41^ but if a lesion is sufficiently small its SUV will not reflect that value.^23^ In our investigation the observed maximum SUV averaged 2.4 ± 0.3 for 25‐mm cylinders and was close to the intended cylinder‐to‐background radioactivity concentration ratio of 2.5,^19^ but was only 1.4 ± 0.2 for 8‐mm cylinders, due to partial volume effects (Table 4). It is not surprising, therefore, that SUV values were not as helpful as other image metrics for detecting small lesions in our lesion simulations. Instead of using maximum SUVs, peak SUVs have the advantage of sampling more voxels and “smoothing out” noise to some extent, but can be imprecise due to the uncertainty of the definition of the most appropriate ROI radius,^37^ especially for lesions that are not as metabolically active as other lesions, and for those lesions that may be metabolically active but small. Corrections to SUV values for partial volume effects have been found to help in assessing metastatic disease,^42^ which can be implemented on PET data only,^9^ but which usually require independent anatomic volume measurements,^43^ such as for those provided by CT, which can be challenging to obtain reliably for small lesions. Furthermore, background activity can be high in normal tissue such as liver, and while trying to identify lesions in the liver by setting an appropriate SUV threshold is one approach that has been used to compute total metabolic lesion volume,^16^ it may be more successful in solitary large tumors than in cases of multiple smaller hepatic lesions.

Our investigation focused on distinguishing a genuine volume of uptake from a similar‐sized uniform radioactivity concentration. Of course, not all small lesions are malignant. If deployed for analyzing clinical studies, a potential extension of our methods would be to first establish that there is a significant likelihood that a volume contains a genuine abnormality, and then to apply additional texture analysis metrics tailored to predict if it will become malignant.^17^, ^44^

An auxiliary benefit of our investigation was to establish which of the studied image metrics best corresponds to the visual impression of the phantom “hot” cylinder visibility. This will be useful for quantifying routinely acquired PET phantom results. Image metrics are more reproducible than visual impressions in quantifying SPECT phantom cold sphere visibility to measure contrast,^24^ and rod visibility to gauge tomographic spatial resolution.^45^ Quantitative “hot” cylinder PET phantom assessment can mitigate potential problems with inter‐observer disagreements, image monitor display setting variability and grayscale choices, and provide a more concrete approach to optimizing reconstruction parameters, and in assessing the success of adjustments to the scanner following maintenance and software upgrades, compared to visual impressions.

Many of the metrics had accuracy over 90% to agree with visualization of “hot” inserts (Table 1) and to discriminate “hot” inserts from the background (Table 2). That is understandable given the obviously high signal‐to‐noise of the three larger “hot” inserts when acquired according to recommended guidelines (Figure 1). Q‐Q curve, polynomial‐fitted, Gaussian‐fitted, and SUV metrics all performed well for the tasks of computing image metrics that agreed well with visual impressions of the larger, “brighter,” phantom inserts and the discrimination of these from background regions.

More challenging was the emulation of visual readings of sub‐centimeter simulated lesions (Table 3) and the discrimination of these from comparable background regions (Table 4). While quantile‐quantile curves (Figure 6) had slopes and intercepts that agreed as well with visual impressions with accuracy comparable to polynomial SNR and contrast (ROC AUC = 87% versus 85–86%) (Table 3), these Q‐Q metrics did not perform nearly as well in discriminating sub‐centimeter inserts from background compared to polynomial‐fit metrics (ROC AUC = 74–75% versus 93–94%) (Table 4). Most notable was the fact that polynomial‐fitted contrast exhibited an accuracy of 93% to perform this discrimination, significantly higher than the 76% accuracy of visual detection and the 67% accuracy of using maximum SUVs. Maximum SUVs have been the main quantitative parameter used to supplement radiologists’ visual interpretations of PET scans,^2^, ^4^ so to have a metric that is more accurate than visual impressions for differentiating lesions from background noise is potentially useful clinically.

Regardless of which of the newer approaches are employed to correct for radiation scatter, denoising, and partial volume effects to PET data,^5^, ^8^, ^9^ including dynamic PET acquisitions,^12^ the resulting set of images must be visually assessed by a physician; based on our phantom results for sub‐centimeter simulated lesions, polynomial‐fit contrast, and polynomial‐fit SNR agreed well with the visual impression of feature visibility (Table 3). Coupled with the fact that these metrics also provided the most accurate discrimination of sub‐centimeter simulated lesions from background ROIs (Table 4), we recommend using polynomial‐fit contrast and polynomial‐fit SNR in evaluating PET QA phantoms and in aiding physicians in deciding whether a small suspicious region of possibly increased tracer uptake is a genuine lesion or background noise.

### 4.1 Limitations

The input data to our algorithms were obtained from different PET/CT scanners and reconstructed by different algorithms, as recommended by the manufacturers of each type of machine. Considering that we had 65 PET scans acquired among 8 different PET systems, we did not have sufficient power to conduct statistically meaningful analyses of possible harmonization or batch effects in our data. Multiple technologists were involved in filling the phantoms and acquiring data at multiple sites. While this had the advantage of providing a range of scans to analyze, there are merits to collecting data in a more regimented fashion at a single site with a single device when analyzing input data, particularly in establishing an ideal baseline set of conditions against which abnormalities are to be detected.

The phantom studies employed in our investigation are signal‐known‐exactly and background‐known‐exactly. While there are some clinical situations that are similar to this, many are not. In practice, there is considerable variability among clinical settings as to imaging noise levels, lesion SUVs, lesion shapes, etc. The simplistic simulations we employed probably would be more successfully applied to small, isolated lesions embedded in uniformly radioactive tissue, such as in the interior of the liver, rather than for lesions on the edge of the liver where larger, abrupt background count changes are expected. Techniques remain to be developed to apply polynomial‐fit contrast and polynomial‐fit SNR approaches to lesions embedded within normal tissue with steep radioactivity concentration gradients.

## 5 CONCLUSIONS

Image texture analysis metrics connect visual impressions of small lesion visibility and are more accurate than visual impressions for detecting sub‐centimeter simulated lesions. Therefore, image texture analysis metrics are potentially clinically useful for ^18^F‐FDG PET/CT studies. In light of the fact that contrast and signal‐to‐noise metrics by polynomial curve fitting provided the highest accuracy to discriminate small, simulated lesions from background image noise, and that Gaussian‐fitting failed for some of the smaller lesions while polynomial‐fitting algorithms converged for all simulated lesions and all background count samples, polynomial curve fitting is the recommended approach to identifying sub‐centimeter lesions in PET scans.

## CONFLICT OF INTEREST

The authors have no conflicts of interest to report.

## AUTHOR CONTRIBUTIONS

All authors contributed significantly to the design, execution, interpretation, and reporting of results for this investigation.

## Data Availability

The data that support the findings of this study are available from the corresponding author upon reasonable request.
